# Supplementary material for: Enhanced auditory disembedding in an interleaved melody recognition test is associated with absolute pitch ability
Source: Sci Rep. 2019 May 24;9:7838. doi: 10.1038/s41598-019-44297-x (PMC6534562; doi:10.1038/s41598-019-44297-x)
Supplement: Supplementary file 1 — Supplementary Material [file 41598_2019_44297_MOESM1_ESM.docx]

**Supplementary Material**

**Enhanced auditory disembedding in an interleaved melody recognition test is associated with absolute pitch ability**

**Teresa Wenhart^1,2^ , Ye-Young Hwang^1^, Eckart Altenmüller^1,2^***

**A**

| **Table A.1.** IMRT differences: imperfect version (N=21) vs. correct version (N=38) | | | | | | | | | |
| --- | --- | --- | --- | --- | --- | --- | --- | --- | --- |
|  | **Version A** | | **Version B** | | | **t-test** | | |  |
|  | *Mean* | *SD* |  | *Mean* | *SD* | | *t* (df) | *p*-value |  |
| All | 3.240 | 0.86 |  | 3.354 | 0.99 | | 0.444 (36.8) | 0.660 |  |
| *0 ST* | 2.04 | 1.21 |  | 2.20 | 1.17 | | -0.488 (42.8) | 0.628 |  |
| *6 ST* | 2.98 | 0.55 |  | 2.99 | 0.80 | | -0.060 (30.7) | 0.952 |  |
| 12 ST | 3.450 | 0.38 |  | 3.458 | 0.38 | | -0.215 (41.0) | 0.941 |  |
| *24 ST* | 3.543 | 0.28 |  | 3.526 | 0.36 | | 0.189 (33.9) | 0.851 |  |
| Means, standard deviations (SD) and statistical comparisons (post-hoc t-test, two tailed) of IMRT performance (perceptual sensitivity index d’) by version (Version A vs. B) and separation condition (in semitones, ST). * p <.05, ** p<.01, . p<.10 | | | | | | | | | |


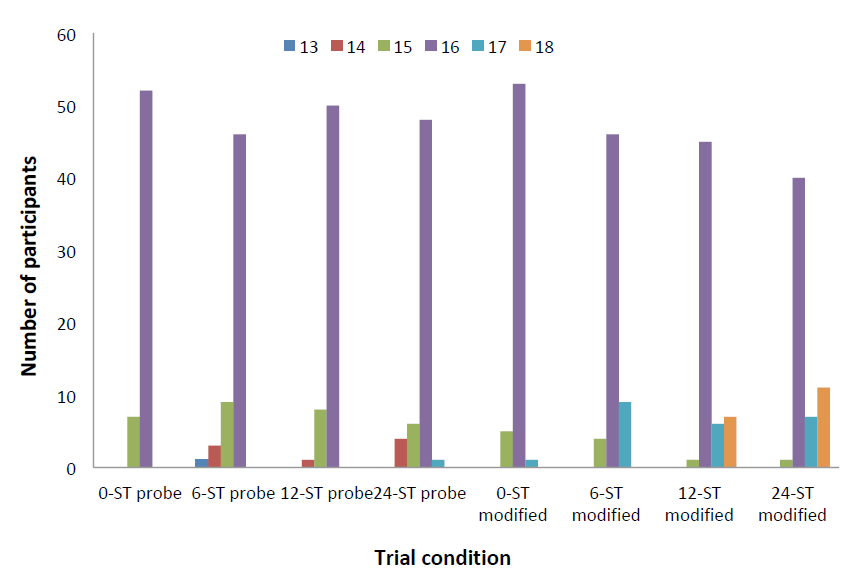


**Figure A.1.** Trial distribution per condition due to randomization error. The figure visualizes the distribution of number of trials for each of the 8 melody conditions (0, 6, 12, 24 ST separation x original (probe)/modified melody (modified)) in the interleaved melody recognition test (IMRT). Due to a technical error regarding randomization of trails the expected number of trials within condition (N=16) was accidentally shifted in N=21 participants. While the total amount of trials remained the same, the number of trials per condition was not (range= 13-18). The distributions per condition show the number of participants receiving 13 to 18 trials within the condition. It can be seen that most of the participants still received the expected 16 trials and of the remaining participants the discrepancy was usually no more than 2 trials.

**B**

| **Table B.1.** IMRT differences: Version A vs. B (N=59) | | | | | | | | | |
| --- | --- | --- | --- | --- | --- | --- | --- | --- | --- |
|  | **Version A** | | **Version B** | | | **t-test** | | |  |
|  | *Mean* | *SD* |  | *Mean* | *SD* | | *t* (df) | *p*-value |  |
| All | 3.000 | 0.8 |  | 3.575 | 0.92 | | -2.586 (55.4) | 0.012 * |  |
| *0 ST* | 1.698 | 1.21 |  | 2.515 | 1.03 | | -2.796 (56.1) | 0.007 * |  |
| *6 ST* | 2.889 | 0.67 |  | 3.084 | 0.62 | | -1.165 (56.9) | 0.249 |  |
| 12 ST | 3.356 | 0.41 |  | 3.554 | 0.31 | | -2.098 (54.0) | 0.041 |  |
| *24 ST* | 3.461 | 0.37 |  | 3.616 | 0.21 | | -1.981 (45.5) | 0.054 |  |
| Means, standard deviations (SD) and statistical comparisons (t-test, two tailed) of IMRT performance (perceptual sensitivity index d’) by version (Version A vs. B) and separation condition (in semitones, ST). Significance after Bonferroni-Holm-correction: * p<.05, ** p<.01 | | | | | | | | | |

| **Table B.2.** IMRT group differences: response bias c (N=59) | | | | | | | | | | |
| --- | --- | --- | --- | --- | --- | --- | --- | --- | --- | --- |
|  | **AP** | | **RP** | | | **t-test** | | |  |  |
|  | *Mean* | *SD* |  | *Mean* | *SD* | | *t* (df) | *p*-value | Cohen´s *d* |  |
| All | 0.093 | 0.29 |  | 0.159 | 0.45 | | 0.677 (53.9) | 0.501 | 0.171 |  |
| *0 ST* | 0.236 | 0.51 |  | 0.474 | 0.78 | | 1.416 (53.8) | 0.163 | 0.357 |  |
| *6 ST* | -0.048 | 0.22 |  | -0.073 | 0.45 | | -0.276 (46.6) | 0.784 | -0.068 |  |
| 12 ST | -0.018 | 0.18 |  | 0.008 | 0.16 | | 0.561 (52.8) | 0.577 | 0.148 |  |
| *24 ST* | 0.005 | 0.16 |  | 0.027 | 0.19 | | 0.469 (56.9) | 0.641 | 0.121 |  |
| Means, standard deviations (SD) and statistical comparisons (post hoc t-test, two-tailed) of response bias c on IMRT by group (AP, absolute pitch; RP, relative pitch) and per separation condition (in semitones, ST). Positive values of *c*  indicate a tendency towards “no” responses and negative values a tendency towards “yes” responses. | | | | | | | | | | |
